# Translating Kratom-Drug Interactions: From Bedside to Bench and Back

**DOI:** 10.1124/dmd.122.001005

**Published:** 2023-08

**Authors:** Rakshit S. Tanna, Nadja B. Cech, Nicholas H. Oberlies, Allan E. Rettie, Kenneth E. Thummel, Mary F. Paine

**Affiliations:** Department of Pharmaceutical Sciences, College of Pharmacy and Pharmaceutical Sciences, Washington State University, Spokane, Washington (R.S.T., M.F.P.); Department of Chemistry and Biochemistry, University of North Carolina at Greensboro, Greensboro, North Carolina (N.B.C., N.H.O.); Center of Excellence for Natural Product Drug Interaction Research, Spokane, Washington (N.B.C., N.H.O., A.E.R., K.E.T., M.F.P.); Departments of Medicinal Chemistry (A.E.R.) and Pharmaceutics (K.E.T.), School of Pharmacy, University of Washington, Seattle, Washington

## Abstract

Kratom is a botanical natural product belonging to the coffee family, with stimulant effects at low doses and opioid-like effects at higher doses. During the last two decades, kratom has been purported as a safer alternative to pharmaceutical and illicit drugs to self-manage pain and opioid withdrawal symptoms. Kratom alkaloids, typically mitragynine, have been detected in biologic samples from overdose deaths. These deaths are often observed in combination with other drugs and are suspected to result from polyintoxications. This review focuses on the potential for kratom to precipitate pharmacokinetic interactions with object drugs involved in these reported polyintoxications. The legal status, chemistry, pharmacology, and toxicology are also summarized. The aggregate in vitro and clinical data identified kratom and select kratom alkaloids as modulators of cytochrome P450 (P450) enzyme activity, notably as inhibitors of CYP2D6 and CYP3A, as well as P-glycoprotein–mediated efflux activity. These inhibitory effects could increase the systemic exposure to co-consumed object drugs, which may lead to adverse effects. Collectively, the evidence to date warrants further evaluation of potential kratom-drug interactions using an iterative approach involving additional mechanistic in vitro studies, well designed clinical studies, and physiologically based pharmacokinetic modeling and simulation. This critical information is needed to fill knowledge gaps regarding the safe and effective use of kratom, thereby addressing ongoing public health concerns.

**SIGNIFICANCE STATEMENT:**

The botanical kratom is increasingly used to self-manage pain and opioid withdrawal symptoms due to having opioid-like effects. The legal status, chemistry, pharmacology, toxicology, and drug interaction potential of kratom are reviewed. Kratom-associated polyintoxications and in vitro-in vivo extrapolations suggest that kratom can precipitate pharmacokinetic drug interactions by inhibiting CYP2D6, CYP3A, and P-glycoprotein. An iterative approach that includes clinical studies and physiologically based pharmacokinetic modeling and simulation is recommended for further evaluation of potential unwanted kratom-drug interactions.

## Introduction

The kratom plant (*Mitragyna speciosa* Korth.) is one of ten species from the *Mitragyna* genus belonging to the coffee family (Rubiaceae) (Brown et al., 2017; Ramanathan et al., 2021). Kratom has been valued for multiple purported medicinal properties since at least the 19th century (Field, 1921). Native to Southeast Asia, this tropical tree is prevalent in Thailand (ithang, kratom, thom), Indonesia (keton, kadamba), Malaysia (biak-biak, ketum), Myanmar (beinsa), Philippines (mambog, lugub, polapoput), and Vietnam (giam) (Grundmann et al., 2023). As a long-standing cultural tradition, manual laborers, particularly farmers and fishermen, chew fresh kratom leaves for the stimulant properties to prevent fatigue (Singh et al., 2015). The leaves also are chewed, smoked, and consumed as a tea during religious ceremonies (Singh et al., 2017). Higher quantities of the leaves are used for pain-relieving and relaxing properties. These properties, along with the wide availability and relatively low cost, led to kratom becoming a substitute for opium in some countries (Brown et al., 2017). Other traditional uses of kratom include appetite suppression and treatment of stomach cramps, diarrhea, and diabetes (Warner et al., 2016).

The effects of kratom have been associated with the plant strain, which is visually characterized based on vein color, including white, green, or red (Suwanlert, 1975; Hartley et al., 2022). Vein color changes as the plant ages, from white at the young stage to green and finally to red after maturation (Ngernsaengsaruay et al., 2022). Vein color has been purported to be associated with different pharmacological effects. For example, the white vein variety is used for mild stimulant effects, the green vein variety is used to manage mild to moderate pain, and the red vein variety is used for stronger pain-relieving and sedating effects. Substrains are based on the geographical location of the plant (Sengnon et al., 2023). However, the validity of these claims has not been scientifically evaluated.

Relative to other species, only *M. speciosa* has been extensively cultivated and used for medicinal benefits. The United States market is dominated by plant products imported from Southeast Asia (Cinosi et al., 2015). At low doses (1–5 g of dried leaf powder), kratom is said to have stimulant properties, whereas at higher doses (>10 g of dried leaf powder), opioid-like effects ensue. Terms such as “legal highs” or “herbal highs” give the perception that kratom is a safe alternative to opioids (Anand and Hosanagar, 2022). Sales of these products at affordable prices via internet vendors or at gas stations and smoke/head shops are widespread (Prozialeck et al., 2012; Williams and Nikitin, 2020).

Dried kratom leaf powder is consumed in western countries as a capsule, pill, or tea (Cinosi et al., 2015). The powder is also swallowed with a drink, otherwise known as the “toss and wash method.” Kratom powder is added to smoothies, cocktails, caffeinated beverages, and even cough syrup. Powders artificially fortified with the kratom alkaloids mitragynine and 7-hydroxymitragynine, termed “enhanced kratom,” are also commonly available. As detailed later, both alkaloids are believed to drive the opioid-like effects of kratom, with ∼10-fold lower and ∼10-fold higher potency than morphine, respectively. Some kratom products available in the United States have been suspected to be artificially enriched with 7-hydroxymitragynine by up to 5-fold of the typical content, which may drive abuse potential (Lydecker et al., 2016). Finally, kratom extracts, tinctures, and resins are available that are more concentrated than the regular dried leaf powders and are sold as low-volume products. To mask the unpleasant taste, kratom is infused in a variety of edible goods, akin to cannabis, that are gaining in popularity.

According to a cross-sectional survey, approximately 3.3 million (1.3%) people in the United States report lifetime kratom use. Most kratom users are male (61%), white (82%), and 18–34 years old (55%) (Schimmel et al., 2021). The rampant use of this once-known-to-be-safe traditional medicine, especially in conjunction with pharmaceutical or illicit drugs, may have exacerbated the risks associated with kratom use in western countries. The number of calls to US poison control centers associated with kratom increased more than 50-fold from 2011 to 2017 (Post et al., 2019).

This review begins with the current legal status and regulation of kratom and active phytoconstituents and the chemistry of these phytoconstituents in kratom leaves. Consideration of the preclinical and clinical pharmacology, toxicology, pharmacokinetics, and drug interaction potential of kratom follows. Our objective was to integrate the aggregate kratom-related knowledge to highlight potential risks associated with consuming kratom with drugs. An iterative approach encompassing clinical evaluations and physiologically based pharmacokinetic modeling and simulation is recommended, with the goal of accurately predicting and simulating real-world kratom-drug interactions. Such a thorough evaluation will facilitate the safe and effective use of this increasingly popular natural product.

## Policy and Legal Status

Kratom is either prohibited or heavily regulated in several countries, including Australia, Bhutan, Canada, Denmark, Finland, Ireland, Italy, Japan, Latvia, Lithuania, Malaysia, Myanmar, New Zealand, Poland, Romania, Sweden, and the United Kingdom (Shah et al., 2021). Until 2021, kratom was prohibited in Thailand, where it was regulated under the Kratom Act of 1943 (Kerrigan and Basiliere, 2022). The US Food and Drug Administration (FDA) has not approved kratom as a safe and effective drug for any medical use. As is common with botanical products, kratom products are regulated differently than FDA-approved drugs. Although not recognized as a dietary supplement according to the Dietary Supplement Health and Education Act, the regulation of kratom is not unlike that of dietary supplements (Paine and Roe, 2018; Paine, 2020). That is, kratom products are not required to undergo extensive safety testing prior to marketing. Accordingly, the onus is on the manufacturers and distributors to ensure that their products are safe and appropriately labeled. However, if a public health concern via adulteration, fraudulence, or other violation of the law is suspected, the FDA can inspect manufacturing facilities or monitor already marketed products.

Because kratom engages the same opioid receptors as morphine, the FDA is concerned that it may pose a risk for addiction, abuse, and dependence (Prozialeck et al., 2019). Accordingly, the FDA imposed an import ban on kratom-containing products, first in 2012 and again in 2014. In 2016, citing safety concerns and to prevent imminent hazards to public safety, the US Drug Enforcement Administration (DEA) announced its intention to place mitragynine and 7-hydroxymitragyine into Schedule I of the Controlled Substance Act of 1970. In response, more than 20,000 kratom advocates, which included users, vendors, and congresspersons, filed comments in the Federal Register (Docket ID: DEA-2016-0015) supporting the usefulness of kratom for the self-treatment of pain and opioid withdrawal symptoms with low potential for abuse. This public outcry contributed to the DEA subsequently withdrawing their decision and listing kratom as a “Drug of Concern.” However, there are several county and statewide (Alabama, Arkansas, Indiana, Rhode Island, Vermont, Wisconsin, and Washington, DC) regulations and/or bans against the possession and use of kratom.

In 2017, the FDA recommended “more research to better understand kratom’s safety profile, including the use of kratom combined with other drugs” to make an informed decision about the regulation of kratom (https://www.fda.gov/news-events/press-announcements/statement-fda-commissioner-scott-gottlieb-md-agencys-scientific-evidence-presence-opioid-compounds). The legal status of kratom within and outside the United States is constantly changing, with increasing kratom-related adverse events and scientific literature reports highlighting the benefit-to-risk ratio associated with kratom use. Kratom has been under Expert Committee on Drug Dependence surveillance since 2020 in response to reports suggesting the potential for abuse, dependence, harm to public health, and fatalities associated with kratom use. However, due to a lack of sufficient evidence about the abuse or dependence potential in humans, the committee recommended against a critical review of kratom and that it instead be kept under surveillance by the World Health Organization secretariat (https://apps.who.int/iris/handle/10665/352462). Continued, fast-paced research on the safety and efficacy of kratom is needed to enable informed decisions about the regulation of kratom.

## Chemistry

**Fig. 1.**
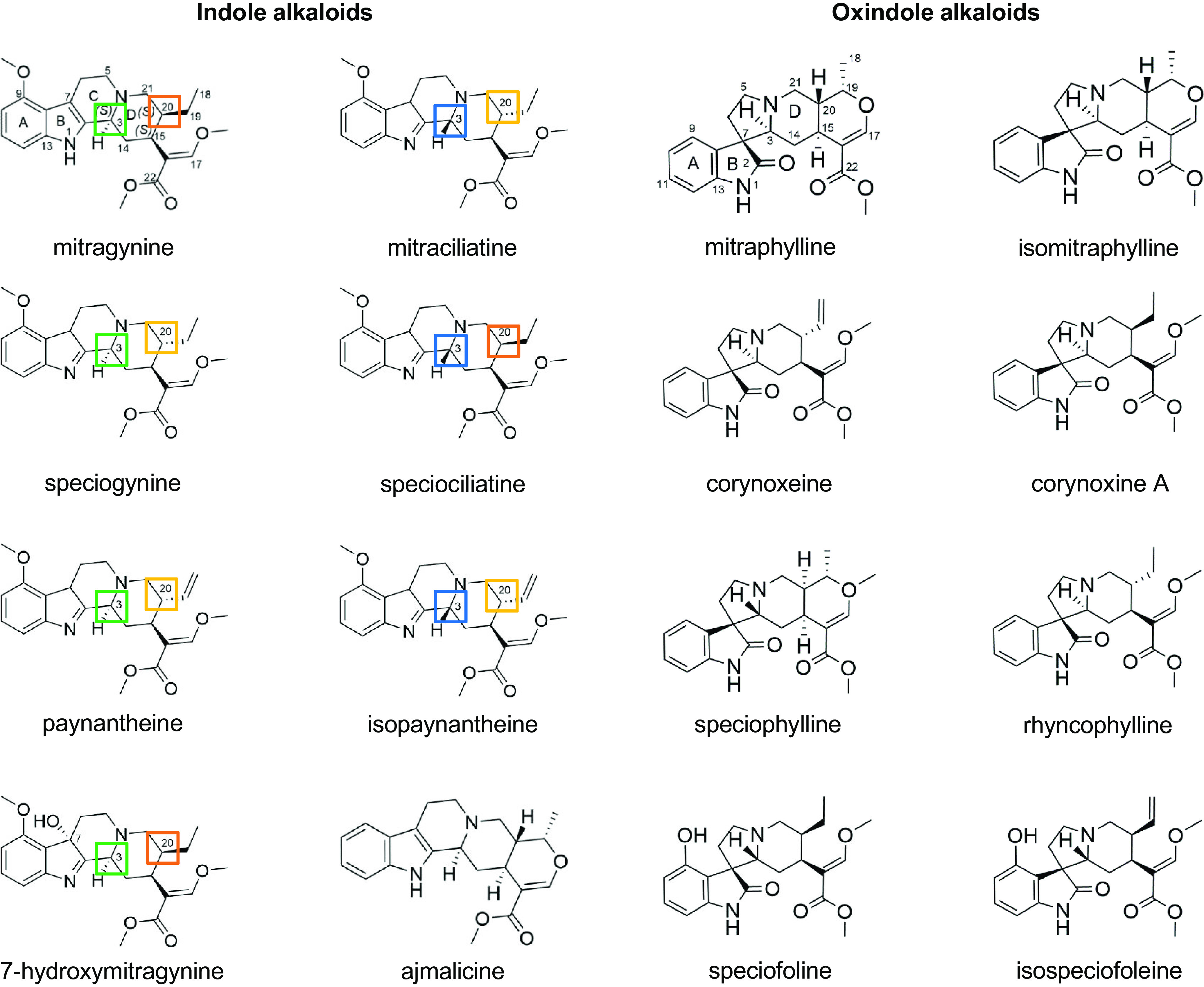
Chemical structures of key indole and oxindole kratom alkaloids. Structures of mitragynine and mitraphylline are numbered to serve as references for the indole and oxindole alkaloids, respectively. Stereochemical differences at C3 and C20 of the indole alkaloids are indicated by the colored boxes. These differences may explain the pharmacokinetic differences observed between those with the 3*S* configuration (mitragynine, speciogynine, paynantheine) and the 3*R* configuration (mitraciliatine, speciociliatine, isopaynantheine) in human adult participants.

Kratom has been investigated extensively for its phytochemical constitution due to having antinociceptive and psychoactive properties. The chemical composition of kratom encompasses alkaloids, terpenoids, flavonoids, tannins, saponins, and phenols. Alkaloids (i.e., basic nitrogen-containing organic phytochemicals) in the kratom plant have been studied predominantly for their pharmacological effects. Total alkaloid content in leaves ranges from 0.5% to 1.5% of dried plant material (Hassan et al., 2013). The alkaloid content and composition in kratom can vary based on the plant’s age, location, or other environmental factors, as well as processing methods. The biosynthesis of alkaloids occurs via a typical indole alkaloid synthesis process, beginning from the shikimic acid pathway combined with the methyl-erythritol phosphate pathway to form corynanthe-type alkaloids (Karunakaran et al., 2022). The kratom plant produces at least 54 alkaloids (Flores-Bocanegra et al., 2020). These alkaloids are largely classified based on the presence of either a tetracyclic indole or pentacyclic oxindole nucleus (Fig. 1). Rings A and B of the tetracyclic indole alkaloids are aromatic and therefore planar in structure, but conformational flexibility is possible in rings C and D (Kerrigan and Basiliere, 2022). The spatial arrangement of these alkaloids has been reported to be influenced by the position of the lone pair of electrons on the nitrogen shared by rings C and D in relation to the exocyclic ethyl/vinyl group on the tetracycle.

Mitragynine (Fig. 1; Table 1) is the major alkaloid in some of the kratom cultivars that are most widely used medicinally (Takayama, 2004). The pharmacological activity of kratom has been attributed largely to mitragynine, along with 7-hydroxymitragynine, which is also formed in vivo via P450-mediated metabolism (Kruegel and Grundmann, 2018; Kruegel et al., 2019; Tanna et al., 2022). Previous studies failed to detect 7-hydroxymitragynine in fresh kratom leaves, leading to the hypothesis that its presence in marketed kratom products may be due to chemical oxidation (autoxidation) of mitragynine during the postharvest phase (Ponglux et al., 1994; Chear et al., 2021).

**TABLE 1:** Typical alkaloidal content in kratom products and dried leaves

Alkaloid	Range of Alkaloid Content*^a^* (mg/g of Product)
mitragynine	0.5–270
speciogynine	3.18–33.4
mitraciliatine	0.647–4.75
speciociliatine	0.185–41.7
paynantheine	1.46–70.4
isopaynantheine	0.269–3.80
7-hydroxymitragynine	0.124–1.10
speciofoline	0.122–5.90
corynoxeine	0.290–1.22
corynoxine A	0.205–11.4
ajmalicine	0.192–0.648
rhynchophylline	0.239

*^a^*Based on two powdered plant products, a loose-leaf product, a liquid product, and an encapsulated powder (Manwill et al., 2022).

Researchers have investigated the pharmacological potential of other kratom alkaloids. Indole alkaloids contain three chiral centers (C3, C15, C20), resulting in multiple prominent mitragynine diastereoisomers that include speciogynine, mitraciliatine, and speciociliatine (Takayama, 2004) (Fig. 1; Table 1). This stereochemistry is responsible for the spatial arrangement of alkaloids, which has been postulated to account for differences in the physicochemical and pharmacological properties among the alkaloids. Related alkaloids include the mitragynine analogs paynantheine and isopaynantheine, which contain a vinyl group instead of an ethyl group at C20. Mitragynine, speciogynine, paynantheine, and 7-hydroxymitragynine possess the 3*S* configuration, whereas speciociliatine, mitraciliatine, and isopaynantheine possess the 3*R* configuration.

Kratom has multiple chemotypes reflective of its geographical origin (Manwill et al., 2022). Metabolomic analysis of more than 50 commercial kratom products revealed at least two different chemotypes predominantly containing either indole or oxindole alkaloids (Fig. 1). These differences led to a wide range of individual alkaloidal content (Todd et al., 2020) (Table 1). Given this reported variability, thorough characterization and appropriate labeling of marketed products are needed to inform the pharmacology, toxicology, or drug interaction potential of kratom. Methods that can be used to characterize botanical and other natural products are described elsewhere (Kellogg et al., 2019).

## Pharmacology

The pharmacology of kratom that elicits the stimulant effects at low doses and relaxing/sedative effects at higher doses remains equivocal. Kratom alkaloids, along with their metabolites, including 7-hydroxymitragynine, bind and act differentially at the opioid, adrenergic, and serotonergic pathway receptors (Table 2). In vitro assays characterizing the binding affinities and functional activities of the alkaloids, followed by the in vivo functional evaluations of the individual alkaloids and leaf extracts, are described below.

**TABLE 2:** Binding affinities (K_i_) of kratom alkaloids toward opioid (OR), adrenergic (*α*), and serotonergic (5-HT) receptors

Receptor	Mitragynine	7-Hydroxy-Mitragynine	Speciogynine	Paynantheine	Speciociliatine	Corynantheidine	Corynoxeine	Corynoxine B	Speciofoline
	*K_i_* ± S.E.M. (nM)								
*μ*OR	238 ± 28 233 ± 48 161 ± 10	16 ± 1 47 ± 18 7.16 ± 0.94	728 ± 61	410 ± 152	54.5 ± 4.4 560 ± 168	118 ± 12	16.3 ± 1.4 >10,000	109.8 ± 8.1 1600	>10,000
*κ*OR	482 ± 29 772 ± 207 198 ± 30	113 ± 37 188 ± 38 74.1 ± 7.8	3200 ± 360 116 ± 36	2560 ± 370	116 ± 36 329 ± 112	1910 ± 50	>10,000	>10,000	>10,000
*δ*OR	>10,000	137 ± 21 219 ± 41	>10,000	>10,000	>10,000	>10,000	>10,000	7600	>10,000
5-HT_1A_	5880 ± 828	—	95.5 ± 34.7	71.8 ± 13.2	—	—	—	—	—
5-HT_7A_	>10,000	—	1600 ± 82	870 ± 72	—	—	—	—	—
5-HT_2A_	5010 ± 1150	—	1320 ± 365	815 ± 192	—	—	—	—	—
5-HT_2B_	1260 ± 138	—	23.0 ± 5.7	20.0 ± 2.8	—	—	—	—	—
5-HT_2C_	>10,000	—	5430 ± 922	1770 ± 417	—	—	—	—	—
*α*-_1A_	1340 ± 100	—	—	—	—	>10,000	—	—	—
*α*-_1B_	4770 ± 120	—	—	—	—	>10,000	—	—	—
*α*-_1D_	5480 ± 540	—	—	—	—	41.7 ± 4.7	—	—	—
*α*-_2A_	4720 ± 120	>10,000	—	—	—	>10,000	—	—	—
*α*-_2B_	9290 ± 30	>10,000	—	—	—	>10,000	—	—	—
*α*-_2C_	2320 ± 140	>10,000	—	—	—	>10,000	—	—	—

—, information not available.

### In Vitro

Both mitragynine and 7-hydroxymitragynine have been shown to be partial agonists at the *μ*-opioid receptor based on inhibition of forskolin-stimulated cyclic AMP (cAMP) accumulation (Todd et al., 2020) and antagonists at the *δ* and *κ* opioid receptors using the G-protein bioluminescence resonance energy transfer functional assay (Kruegel et al., 2016). In contrast to other opioids like morphine, mitragynine and 7-hydroxymitragynine selectively activate the G-protein second messenger upon binding to the G-protein–coupled receptor rather than the *β*-arrestin-2 signaling pathway, which is responsible for many of the adverse effects associated with typical opioids. Based on results from the guanosine 5′-O-(3-[^35^S]thio)triphosphate ([^35^S]GTP*γ*S) stimulation assay, mitragynine was reported to be a competitive antagonist at *μ*-opioid receptors (Obeng et al., 2021), suggesting that other alkaloids may contribute to the opioid-like effects. Other kratom alkaloids, such as speciociliatine and mitraciliatine, have been reported to be partial agonists at the *μ*-opioid receptor (Hiranita et al., 2022). Mitragynine has also been shown to engage several nonopioid receptors, including *α*_2_ adrenergic, adenosine A2a, dopamine D2, and the serotonin receptors 5-HT2C and 5-HT7 (Anand and Hosanagar, 2022). Mitragynine was shown to be a partial agonist at *α*_1A,D_ receptors and a competitive antagonist at *α*_1A,B,D,2C_ receptors (Obeng et al., 2020). Corynanthidine, a 9-demethoxy analog of mitragynine, was ∼130-fold more potent than mitragynine at *α*_1A,D_ receptors (Table 2). Mitragynine exhibited low binding affinity to serotonergic receptors, whereas speciogynine and paynantheine demonstrate high binding affinity to the 5-HT1A and 5-HT2B serotonergic receptors (León et al., 2021).

### In Vivo

Kratom extract, tea, and purified alkaloids including mitragynine, 7-hydroxymitragynine, speciociliatine, mitraciliatine, isopaynantheine, corynanthidine, and corynoxeine have shown robust antinociceptive effects in rodents using the hot plate and/or tail-flick assays (Matsumoto et al., 1996a,b; Chin and Mark-Lee, 2018). Upon oral or parenteral administration of mitragynine, a partial antinociceptive effect was observed in rats and mice compared with the full opioid agonist morphine. In contrast, 7-hydroxymitragynine produced a ∼3- to 10-fold more potent antinociceptive effect than morphine (Chakraborty et al., 2021). The observed antinociceptive effects decreased when the *μ*-opioid receptor antagonist naltrexone was administered intraperitoneally, suggesting that these effects are primarily *μ*-opioid receptor mediated. Although 7-hydroxymitragynine is more potent than mitragynine, its role in the overall antinociceptive effect after mitragynine administration is inconclusive, largely due to the lower brain concentration of 7-hydroxymitragynine (Berthold et al., 2022). Mitragynine was shown to have more potent effects in rodent models when administered orally compared with intraperitoneal or subcutaneous routes, suggesting potential first-pass bioactivation of this alkaloid to more potent metabolites (Kruegel and Grundmann, 2018). Overall, the in vivo efficacy of kratom appears to be mediated by mitragynine, other alkaloids, and their metabolites. However, definitive roles of several other alkaloids isolated from the kratom leaves remain uncertain.

One study objectively assessed the effects of kratom on pain tolerance in humans (Vicknasingam et al., 2020). This randomized, placebo-controlled, double-blind study involved 26 healthy male long-term kratom users. A significant increase in pain tolerance in the cold pressor task (∼11 to ∼25 seconds) was reported 1 hour after kratom ingestion. Clinical evaluation of the stimulant effects of kratom at lower doses remains lacking.

## Toxicology

### Nonhuman

After oral administration of a methanolic kratom extract to rats (100–1000 mg/kg) for 14 days, mild nephrotoxicity and moderate to severe hepatotoxicity were observed, with elevations in alanine aminotransferase, aspartate aminotransferase, albumin, triglycerides, and cholesterol (Harizal et al., 2010). After oral administration of a different kratom extract to mice, the LD_50_ (lethal dose, 50%) ranged from ∼170 to 590 mg/kg (Reanmongkol et al., 2007; Sabetghadam et al., 2013a). After oral administration of mitragynine to rats (1–10 mg/kg) for 28 days, no adverse effects were observed (Sabetghadam et al., 2013b). At a higher dose of mitragynine (100 mg/kg), biochemical, hematologic, and histopathological abnormalities involving the liver, brain, and kidney surfaced, but there were no deaths. A single oral dose of mitragynine administered to dogs (80 mg/kg) produced no adverse effects, including respiratory depression (Kruegel and Grundmann, 2018). A subchronic low oral dose of mitragynine administered to dogs (5–20 mg/kg per day) for 21 days also produced no adverse effects; however, after oral administration of a higher dose (40 mg/kg per day) to these dogs for the subsequent 21 days, changes in blood chemistry, liver cell morphology, and lymphatic hyperplasia were observed. Overall, based on the evidence from preclinical species, kratom as an extract and mitragynine as the purified alkaloid may be toxic only with chronic use at high doses.

### Human

Both acute and chronic kratom toxicology have been investigated. Kratom-associated risks and adverse events have been reported based on cross-sectional surveys from active kratom users, national poison data systems, and clinical case reports (Kerrigan and Basiliere, 2022). Common adverse effects include agitation, tachycardia, drowsiness, vomiting, and confusion (Eggleston et al., 2019). Severe adverse effects include hepatotoxicity, cardiotoxicity, respiratory depression, seizure, neonatal abstinence syndrome, hypothyroidism, overdose toxidrome, and fatalities (Alsarraf et al., 2019). Kratom-induced intrahepatic cholestasis has been reported after chronic (∼1–4 weeks) use of large kratom doses (>10 g per day) (Schimmel and Dart, 2020). In some cases, kratom-induced hepatotoxicity was completely reversed after kratom cessation. However, due to polysubstance use, typical with kratom use, the toxicology of kratom remains unclear. According to the FDA Adverse Event Reporting System (FAERS) database, from October 2012 through September 2021, 489 adverse events associated with kratom (i.e., mitragynine) have been reported, including 244 deaths (Li et al., 2023). Most of these deaths were believed to result from co-consumption with other drugs.

Depending on the symptoms, kratom-related overdose has been managed with the *μ*-opioid receptor antagonist naloxone to reverse the opioid-like effects (either from kratom or coingested opioids), whereas the dual *α* and *β* adrenergic antagonist labetalol has been used to manage sympathetic overactivation (Peran et al., 2023). Kratom-related withdrawal symptoms have been managed with a combination of naloxone and the partial *μ*-opioid agonist buprenorphine (Suboxone) (Weiss and Douglas, 2021).

## Pharmacokinetics

The pharmacokinetics of key kratom alkaloids have been characterized in both preclinical species and human participants. Intravenous and oral administration of kratom extracts and individual alkaloids, including mitragynine, have been extensively investigated in rodents and dogs, as reviewed elsewhere (Hiranita et al., 2022). In contrast, only two studies to date have focused on the pharmacokinetics of mitragynine and other kratom alkaloids in humans (Trakulsrichai et al., 2015; Tanna et al., 2022) (Table 3). Regarding the earlier study (Trakulsrichai et al., 2015), blood was collected from 0 to 24 hours, yet an average terminal half-life of ∼24 hours was reported, raising concerns about the accuracy of this and other fundamental pharmacokinetic parameters reported, including oral clearance (CL/F) and apparent volume of distribution (V_d_/F) (Tanna et al., 2022). Accordingly, results from the latter study, where blood was collected from 0 to 120 hours, are presented (Table 3). These latter pharmacokinetic observations in humans are further supported by pertinent in vitro studies (discussed below). The following section summarizes the physicochemical and pharmacokinetic properties of these alkaloids as they may relate to the risk of kratom-drug interactions in humans.

**TABLE 3:** Clinical pharmacokinetics of key kratom alkaloids after oral administration of kratom tea to healthy adult participants

Study	Trakulsrichai et al. (2015)	Tanna et al. (2022)						
Participants (*n*)	10	5						
	Mean ± S.D.	Median (Range)						
Alkaloid	Mitragynine	Mitragynine	Speciogynine	Paynantheine	Speciociliatine	Mitraciliatine	Isopaynantheine	7-Hydroxy-Mitragynine
Dose (mg)	Variable*^a^*	39	6.4	12	10	1.3	1.0	—
C_max_ (nM)	—	81.9 (50.1–177)	51.4 (34.2–121)	61.1 (56.4–157)	308 (154–380)	73.5 (34.9–98.6)	48.8 (26.2–68.2)	16.1 (11.9–22.2)
t_max_ (h)	0.83 ± 0.35	1 (0.75–1.5)	2 (1–3.5)	1 (0.75–2.5)	2.5 (1–3.5)	4.5 (3.5–6.5)	4.5 (2.5–6.5)	1 (0.75–2.5)
t_1/2_ (h)	23 ± 16	45.3 (31.9–50.2)	23.5 (16.1–28.3)	27.0 (17.7–30.8)	12.3 (10.4–21.1)	17.8 (11.2–24.7)	14.4 (11.8–20.9)	5.67 (5.03–6.52)
AUC_inf_ (nMh)	—	420 (324–1360)	477 (379–1120)	438 (389–956)	5120 (3200–7560)	1160 (1040–3520)	794 (667–2130)	106 (60.8–126)
V_z_/F (L)	2900 ± 1850*^b^*	12,700 (5190–19,700)	962 (584–1235)	1940 (1370–2620)	130 (60.1–159)	46.0 (26.2–74.0)	55.5 (36.6–76.0)	—
CL/F (l/h)	7550 ± 3900*^b^*	233 (71.7–302)	33.5 (14.3–42.1)	67.4 (30.9–76.0)	5.01 (3.40–8.04)	2.78 (0.92–3.11)	3.25 (1.21–3.87)	—

—, information not available.

AUC_inf_, area under the plasma concentration-time profiles from time zero to infinity; CL/F, oral clearance; t_1/2_, terminal elimination half-life; T_max_, time to reach C_max_; V_z_/F, apparent volume of distribution during the terminal phase.

*^a^*A range of doses (6.25–23 mg) of mitragynine contained in a kratom tea were administered, precluding direct comparison of C_max_ and AUC_inf_ between studies.

*^b^*Obtained by multiplying the V_d_/F in l/kg with the mean weight (77 kg) of the participants; blood was collected from 0 to 24 hours, yet an average terminal half-life of ∼24 hours was reported, raising concerns about the accuracy of the reported pharmacokinetic outcomes, including t_1/2_, CL/F, and V_z_/F.

### Physicochemical Properties

Mitragynine is categorized as a Biopharmaceutics Classification System class 2 xenobiotic, meaning it has high permeability and low aqueous solubility. Mitragynine and other kratom alkaloids, including 7-hydroxymitragynine, speciogynine, and paynantheine, were shown to have variable stability across a wide range of temperatures and pH (Basiliere and Kerrigan, 2020). In general, the extent of degradation was higher at elevated temperatures (>40°C) and under more acidic (pH 2–4) or alkaline (pH 8–10) conditions. Both mitragynine and 7-hydroxymitragynine degraded by <10% in simulated gastric (pH 1.2) and intestinal (pH 6.8) fluids after 30 minutes and 3 hours, respectively. However, near the physiologic conditions of temperature and pH of the stomach, intestine, tissues, and systemic circulation, the extent of degradation is expected to be more limited. These observations suggest that upon oral administration, the entire doses of the kratom alkaloids administered may be available at the site of absorption. Mitragynine was shown to have an octanol:water partition coefficient (Log P) of 1.7 (Ramanathan et al., 2015) but a higher in silico predicted Log P of 3.4–4.2 using ADMET Predictor (v10.4; Simulations Plus, Inc., Lancaster, CA). The lipophilicity of kratom alkaloids has been reported to be dependent on the stereochemistry at the chiral centers (Trager et al., 1967; Beckett and Dwuma‐Badu, 1969). Alkaloids with the 3*S* configuration (e.g., mitragynine, speciogynine, and paynantheine) were shown to have a planar conformation and to be more lipophilic compared with alkaloids with the 3*R* configuration (e.g., mitraciliatine, speciociliatine, and isopaynantheine) (Fig. 1), which were shown to have a bent conformation. The log ionization constant (pKa) of these alkaloids was also dependent on the stereochemistry but within a narrow range (7.06–7.95).

### Absorption

After oral administration of a kratom tea to six human participants, the key kratom alkaloids were rapidly absorbed and measurable in the systemic circulation (Tanna et al., 2022). The rate of kratom alkaloid absorption was variable, with the maximum plasma concentration (C_max_) achieved within 1–4.5 hours. Kratom indole alkaloids with the 3*S* configuration generally exhibited a shorter time to reach C_max_ (t_max_) than those with the 3*R* configuration (1 to 2 vs. 2.5–4.5 hours). This difference has been attributed to differences in the partition coefficients of the alkaloids (Beckett and Dwuma‐Badu, 1969). The absolute oral bioavailability (F_oral_) of mitragynine in humans is unknown, and preclinical data suggest species-specific differences. For example, the F_oral_ of mitragynine (3%–30%) after administration of 20–50 mg/kg to rats was lower than that in beagle dogs, which exhibited an F_oral_ of ∼70% after a single dose of 5 mg/kg (Parthasarathy et al., 2010; Avery et al., 2019; Maxwell et al., 2020). Based on an allometric method involving the intravenous clearance of mitragynine in beagle dogs and the oral clearance of mitragynine (administered as kratom) in humans (Tanna et al., 2022), human F_oral_ of mitragynine was estimated at ∼30%.

Using the parallel artificial membrane permeability assay (PAMPA), mitragynine flux through the phospholipid bilayer at pH 4 and 7.4 was 0.23 × 10^−6^ and 11 × 10^−6^ cm/s, respectively (Kong et al., 2017). Higher permeability at pH 7.4 is consistent with mitragynine permeating as the unionized form. Using Caco-2 cell monolayers (mimicking the intestinal barrier), absorptive [apical (A)→basolateral (B)] and exsorptive (B→A) flux for mitragynine at 10 *μ*M was 2.5 × 10^−5^ and 2.9 × 10^−5^ cm/s, respectively, suggesting moderate to high permeability (Manda et al., 2014b). The flux ratio [(B→A)/A→B)] was approximately unity, suggesting that mitragynine is not a substrate for the efflux transporter P-glycoprotein (P-gp). Likewise, mitragynine flux through Madin-Darby canine kidney cell monolayers overexpressing multidrug resistance 1 protein (MDR-MDCK) at 10 *μ*M was 16 × 10^−6^ and 18 × 10^−6^ cm/s, respectively, with a flux ratio of 1.1. In contrast, the minor alkaloid mitraphylline was shown to be a substrate for P-gp (flux ratio ∼6 to 7) (Manda et al., 2014b).

### Distribution

After administration of kratom tea to healthy human adult participants, concentration-time profiles for the key kratom alkaloids were best described by a two-compartment model (Tanna et al., 2022). In general, the extent of tissue distribution was higher for the indole alkaloids with the 3*S* configuration compared with those with the 3*R* configuration, as indicated by a larger apparent volume of distribution, specifically a larger apparent peripheral volume of distribution. Alkaloids with the 3*S* configuration were also shown to be rapidly distributed into the peripheral tissues and to redistribute slowly out of the peripheral tissues relative to the alkaloids with the 3*R* configuration. Preclinical pharmacokinetic studies of mitragynine and speciociliatine in rodents reported similar trends in distribution (Berthold et al., 2021).

These differences in alkaloid distribution were again attributed to differences in the lipophilicity of the 3*S* and the 3*R* configured indole alkaloids (Tanna et al., 2022) but could also be due to lysosomal trapping of the alkaloids in various cells, as reported for other cationic lipophilic drugs like chloroquine (Macintyre and Cutler, 1988). Kratom alkaloids are highly bound to plasma proteins, with a fraction unbound of <0.06 at 1 *μ*M (Tanna et al., 2022). Human blood-to-plasma ratios for kratom alkaloids ranged from 0.65 to 1.05 at 1 *μ*M, indicating limited partitioning into erythrocytes. Tissue distribution of mitragynine in mice was perfusion rate limited, wherein the ratio of AUC_organ_ (area under the organ concentration vs. time curve) to AUC_plasma_ was directly proportional to the blood flow of the respective organ (AUC_organ_/AUC_plasma_ in liver, 28.6 > kidney, 17.1 > lung, 15.9 > spleen, 5.1) except the brain due the blood-brain barrier (Yusof et al., 2019; Berthold et al., 2022). The AUC_brain_/AUC_plasma_ for the more polar 7-hydroxymitragynine was <1.

### Elimination

In general, kratom alkaloids demonstrated a long elimination half-life upon administration of kratom tea to healthy adult participants (Tanna et al., 2022). Following the trend observed for absorption and distribution, the terminal half-lives for the indole alkaloids with the 3*S* configuration (24–45 hours) were longer than analogs with the 3*R* configuration (∼12–18 hours). In addition, the fraction of the dose excreted unchanged in the urine (f_e_) was higher for the 3*R* compared with the 3*S* configured alkaloids. However, for all alkaloids, f_e_ was <0.2%, indicating that urinary excretion is a minor route of systemic elimination. Consistent with that interpretation, renal clearance (CL_R_) for all alkaloids was much lower than effective renal plasma flow (<0.5 vs. 36 l/h).

**Fig. 2.**
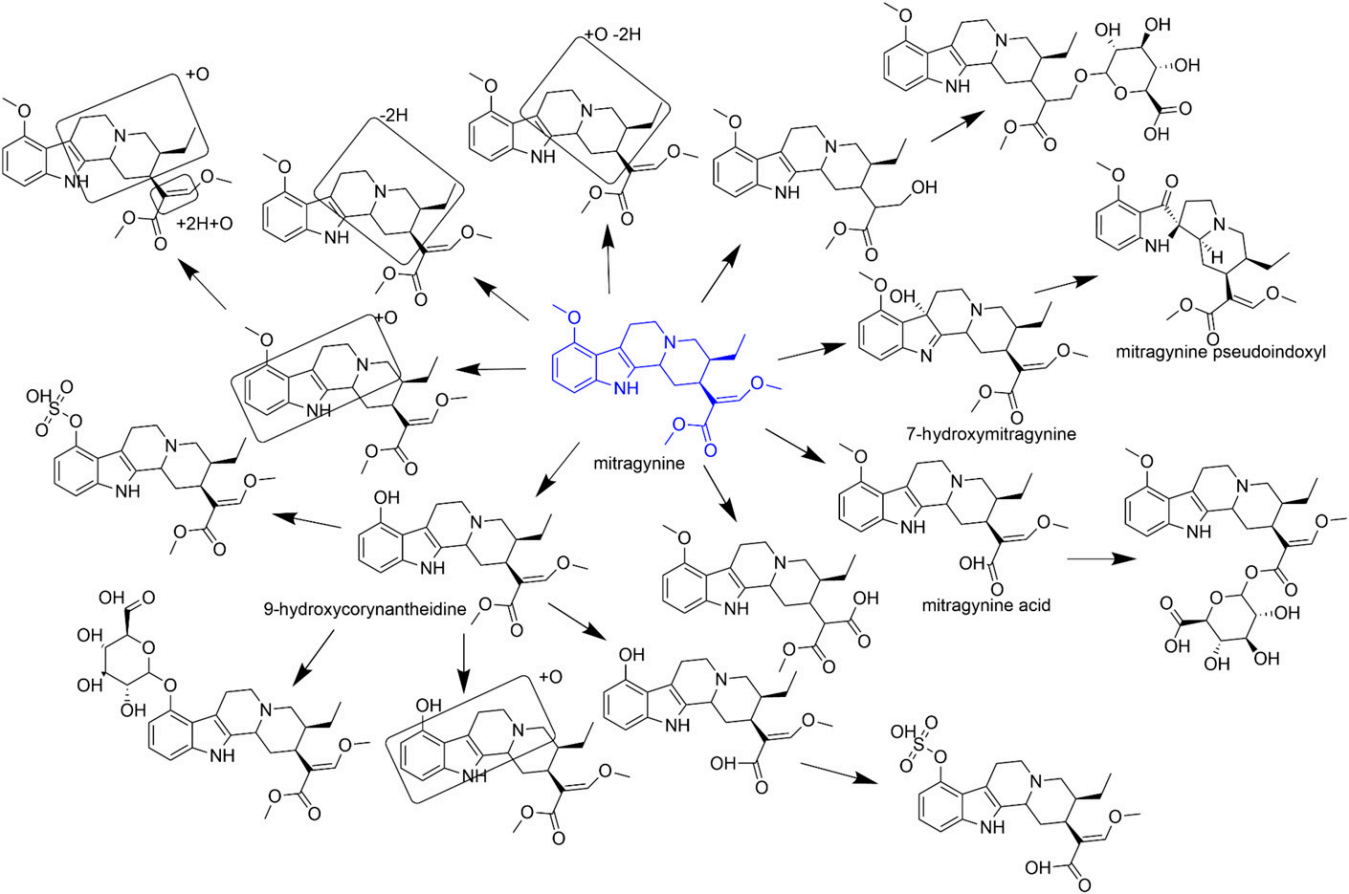
Proposed metabolic scheme for mitragynine based on reported in vitro and in vivo evaluations. Boxes suggest the tentative site of metabolism.

Kratom alkaloids, including mitragynine, primarily undergo oxidative metabolism, which can subsequently be either glucuronidated or sulfated based on the species (Philipp et al., 2009; Basiliere et al., 2018) (Fig. 2). These alkaloids were extensively metabolized in an NADPH-dependent manner in both enteric (human intestinal microsomes) and hepatic (human liver microsomes) tissue fractions (Tanna et al., 2022). The extent of metabolism of kratom alkaloids is stereoselective (i.e., the indole alkaloids with the 3*S* configuration are more rapidly metabolized than those with the 3*R* configuration). Cytochrome P450 (P450) 3A4 is the major enzyme that metabolizes mitragynine to 7-hydroxymitragynine (Kamble et al., 2019; Kruegel et al., 2019). 7-Hydroxymitragynine was further reported to be metabolized by an unknown human plasma enzyme to a 31-fold more potent activator of the *μ*-opioid receptor, mitragynine pseudoindoxyl (Kamble et al., 2020a). CYP2C9, CYP2C19, and CYP2D6 have minor roles in the metabolism of mitragynine (Kamble et al., 2019). Mitragynine acid formation from mitragynine was catalyzed by human carboxylesterase (hCES) 1c (K_m_ = 87 *μ*M; V_max_ = 0.7 nmol/min per mg) but not by hCES2 (Meyer et al., 2015). However, based on the low intrinsic clearance, the clinical relevance of the hCES1c pathway is unlikely.

## Kratom-Drug Interactions

### In Vitro Evidence

Kratom has been extensively evaluated in vitro as a precipitant of pharmacokinetic drug interactions. Methanolic extracts of kratom and purified kratom alkaloids were shown to alter the activity of key drug metabolizing enzymes, including P450s and UDP-glucuronosyltransferases (UGTs) responsible for the elimination of xenobiotics (Table 4). Kratom extracts were initially shown to inhibit CYP1A2, CYP2C19, CYP2D6, and CYP3A using high-throughput fluorometric assays, with IC_50_ ranging from ∼0.6 (CYP2D6) to 140 (CYP3A) *μ*g/ml (Kong et al., 2011). Methanolic extracts prepared from well characterized kratom products were later shown to inhibit CYP2C9 (∼65% at 20 *μ*g/ml), CYP2D6 (∼90% at 20 *μ*g/ml), and CYP3A (∼50% at 20 *μ*g/ml) activities using selective probe drug substrates and a liquid chromatography–mass spectrometry (LCMS)-based assay (Todd et al., 2020). The extent of inhibition remained consistent among three kratom extracts despite differences in alkaloidal content and composition.

**TABLE 4:** IC_50_ values for mitragynine and kratom extracts against P450 and UGT activities using various test systems

Enzyme	Substrate	Reaction	Enzyme System	Monitoring Method	Mitragynine (*μ*M)	Extract (*μ*g/ml)
CYP1A2	phenacetin	*O*-deethylation	HLMs	LCMS	>45	—
	CEC	*O*-deethylation	recombinant	fluorometric	—	39
CYP2C8	amodiaquine	*N*-deethylation	HLMs	LCMS	33.5	—
CYP2C9	diclofenac	4’-hydroxylation	HLMs	LCMS	>45	—
	luciferin H				9.7	—
	diclofenac	4’-hydroxylation	HLMs	LCMS	39.7	—
CYP2C19	*S*-mephenytoin	4’-hydroxylation	HLMs	LCMS	10.5	—
	CEC	*O*-deethylation	recombinant	fluorometric	—	85
CYP2D6	dextromethorphan	*O*-demethylation	HLMs	LCMS	2.2	—
	AMMC	*O*-demethylation	recombinant	fluorometric	—	0.64
	luciferin ME-EGE			luminometric	0.45	—
	luciferin ME-EGE			luminometric	—	3.6
	dextromethorphan	*O*-demethylation	HLMs	LCMS	0.67	—
CYP3A4/5	Midazolam	1’-hydroxylation	HLMs	LCMS	11.4	—
	testosterone	6*β*-hydroxylation	HLMs	LCMS	>45	—
	BFC	*O*-debenzylation	recombinant	fluorometric	—	0.78
	midazolam	1’-hydroxylation	recombinant	HPLC-UV	17.31	—
	testosterone	6*β*-hydroxylation	recombinant	HPLC-UV	3.98	—
	luciferin-BE			luminometric	41.32	—
	luciferin-BE			luminometric	—	142.8
	midazolam	1’-hydroxylation	HLMs	LCMS	18.9	—
	midazolam	1’-hydroxylation	HIMs	LCMS	21.9	—
UGT1A1	4-MU	glucuronidation	recombinant	HPLC-UV	>100	—
UGT2B7	zidovudine	glucuronidation	HLMs	HPLC-UV	8.11	—
	4-MU	glucuronidation	recombinant	HPLC-UV	>100	—

—, information not available.

AMMC, 3-[2-(N,N-diethyl-N-methylammonium)ethyl]-7-methoxy-4-methylcoumarin; BFC, 7-benzyloxy-4-(trifluoromethyl)-coumarin; CEC, 3-cyano-7-ethoxycoumarin; HPLC-UV, high pressure liquid chromatography coupled with UV detector; luciferin-BE, luciferin 6’ benzyl ether; luciferin H, 6’deoxyluciferin; luciferin ME-EGE, ethylene glycol ester of luciferin 6’ methyl ether; 4-MU, 4-methylumbelliferone.

Mitragynine inhibited the activity of several P450 enzymes in human liver microsomes (HLMs), including CYP2C8, CYP2C9, CYP2C19, CYP2D6, and CYP3A, with IC_50_ values ranging from 0.45 *μ*M (CYP2D6) to ∼40 *μ*M (CYP2C9) (Hanapi et al., 2013; Lim et al., 2013; Kamble et al., 2020b) (Table 4). Inhibition kinetics alone do not necessarily indicate the risk of a pharmacokinetic kratom-drug interaction. The likelihood of an interaction depends on several factors, including route of administration, dose of the precipitant, enzyme expression, extent of plasma protein binding, and disposition of the precipitant. Using basic static models recommended by the FDA, a 40-mg oral dose of mitragynine (contained in a 2-g oral dose of kratom) was predicted to precipitate a presystemic intestinal interaction for CYP3A, a presystemic hepatic interaction for CYP2D6, but no systemic interaction (Table 5). No interaction was predicted for any of the other tested P450s. These predictions warrant further investigation of the drug interaction potential of kratom when consumed with drugs metabolized by CYP3A and CYP2D6.

**TABLE 5:** Prediction of the pharmacokinetic drug interaction potential of kratom using mitragynine as the precipitant. A basic static model for reversible inhibition was used to predict the drug interaction potential of mitragynine administered orally via inhibition of cytochromes P450. R values indicate the predicted AUC ratio of the object drug in the presence to absence of inhibitor for intestine (R_1,gut_) or using hepatic (R_1,hep_) and systemic (R_1,sys_) concentration.

Enzyme	IC_50_ (*μ*M)	*K_i_*^a^ (*μ*M)	R_1,gut_	R_1,hep_	R_1,sys_	Reference
CYP1A2	>45	>22.5	NA	1.01	1.00	(Kamble et al., 2020b)
CYP2C8	33.5	16.8	NA	1.01	1.00	(Kamble et al., 2020b)
CYP2C9	39.7	19.9	NA	1.01	1.00	(Tanna et al., 2021)
CYP2C19	10.5	5.25	NA	1.04*	1.00	(Kamble et al., 2020b)
CYP2D6	0.67	0.34	NA	1.57*	1.01	(Tanna et al., 2021)
CYP3A	11.4	5.7	71*	1.03*	1.00	(Tanna et al., 2021)
P-gp^b^	18.2	—	22*	—	—	(Manda et al., 2014a)

—, information not available.

^a^K_i_ values are estimated as IC_50_/2 using the Cheng-Prusoff equation assuming competitive inhibition.

^b^R_1,gut_ = 1 + (I_gut_/K_i_) or (I_gut_/IC_50_) for P-gp, where intestinal luminal concentration (I_gut_) was calculated as dose/250 ml (∼400 μM).

*R_1,gut_ ≥11 and R_1,hep_ or R_1,sys_ ≥1.02 indicates a potential interaction. R_1,hep_ = 1 + (I_hep,u_/K_i_), where I_hep,u_ was calculated as f_u,p_ × (C_max_ + F_a_ × k_a_ × dose/Q_h_/R_B_) (∼0.19 μM); R_1,sys_ = 1 + (I_sys,u_/K_i_), where I_sys,u_ is f_u,p_ × C_max_.

Mitragynine caused an ∼7-fold leftward shift in IC_50_ toward CYP3A activity upon a 30-minute preincubation with NADPH (Tanna et al., 2021) and was confirmed to be a time-dependent inhibitor of CYP3A activity in both HLMs [time-dependent inhibition constant (*K_I_*) = 4.1 ± 0.9 *μ*M; maximum rate of inactivation (*k_inact_*) = 0.068 ± 0.01 min^−1^] and human intestinal microsomes (HIMs) (4.2 ± 2.5 *μ*M; *k_inact_* = 0.079 ± 0.02 min^−1^) (Table 6). The mechanism of the observed time-dependent inhibition was not ascertained. Such NADPH-dependent inhibition over time is most commonly attributed to P450-mediated bioactivation of the compound, generating a reactive species that irreversibly binds to the enzyme, rendering the enzyme inactive. Although no direct structural alerts are present in mitragynine, based on literature evidence gathered for compounds with similar structural features as mitragynine, it was speculated that bioactivation of the quinolizidine moiety to an imine, 3-methylindolenine, *p*-quinone, or *o*-quinoneimine intermediate may result in covalent binding to enzyme nucleophilic residues (Tanna et al., 2021). Other compounds, including evodiamine, rutaecarpine, and zafirlukast, have been shown to inactivate CYP3A4 via a 3-methylindolenine–like intermediate, rendering this mechanism the most probable. Further in vitro evaluation is needed to confirm the exact mechanism. No IC_50_ shift was observed for the inhibition of CYP2C9 and CYP2D6 activities. However, strong inhibition of CYP2D6 activity was observed and of a reversible competitive nature (*K_i_* ∼1.2 *μ*M) (Table 6).

**TABLE 6:** Inhibition kinetics for mitragynine against P450 activities

Enzyme	Substrate	Reaction	Enzyme System	Monitoring Method	Mode of Inhibition	Inhibition Kinetics
CYP2C9	luciferin H			luminometric	noncompetitive	K_i_ = 155 *μ*M
CYP2D6	dextromethorphan	*O*-demethylation	HLMs	LCMS	competitive	K_i_ = 1.17 *μ*M
	dextromethorphan	*O*-demethylation	HLMs	LCMS		K_i_ = 1.1 *μ*M
	luciferin ME-EGE			luminometric	noncompetitive	K_i_ = 12.86 *μ*M
CYP3A4/5	midazolam	1’-hydroxylation	HLMs	LCMS	time dependent	K_I_ = 4.1 *μ*M; k_inact_ = 0.068 min^−1^
	midazolam	1’-hydroxylation	HIMs	LCMS	time dependent	K_I_ = 4.2 *μ*M; k_inact_ = 0.079 min^−1^
	luciferin-BE			luminometric	competitive	K_i_ = 380 *μ*M

luciferin-BE, luciferin 6’ benzyl ether; luciferin H, 6’deoxyluciferin; luciferin ME-EGE, ethylene glycol ester of luciferin 6’ methyl ether.

An in vitro to in vivo extrapolation (IVIVE) method was used to predict the likelihood of P450-mediated pharmacokinetic drug interactions upon oral consumption of a low dose (2 g) of kratom. Using an established mechanistic static model for time-dependent inhibition, a high interaction risk was predicted with the CYP3A probe substrate midazolam, as indicated by an AUCR (area under the plasma concentration vs. time curve in the presence to absence of inhibitor) of 5.7. Such a high magnitude of interaction can be attributed to the abundant expression of CYP3A in enterocytes coupled with a high mitragynine concentration in the intestinal lumen relative to the liver (Thelen and Dressman, 2009). In contrast, the mechanistic static model using reversible inhibition predicted a low interaction risk with the CYP2D6 probe substrate dextromethorphan (AUCR = 1.1). Although the likelihood of an interaction with CYP2D6 substrates was predicted to be low, clinically relevant interactions may still result at higher kratom doses.

Other alkaloids, including paynantheine, speciogynine, speciociliatine, corynantheidine, and 7-hydroxymitraynine, have been shown to differentially inhibit P450 activities (Kamble et al., 2020b). In general, like mitragynine, these other alkaloids reversibly inhibited CYP2D6 (IC_50_ ∼4–13 *μ*M), CYP3A (IC_50_ ∼7–26 *μ*M), and CYP2C19 (IC_50_ ∼8–38 *μ*M). The extent of inhibition of CYP2C9, CYP2D6, and CYP3A activities by kratom extracts (20 *μ*g/ml) containing 1 *μ*M mitragynine was higher compared with an equimolar concentration of purified mitragynine (1 *μ*M) tested alone (Todd et al., 2020). Mitragynine accounted for most of the inhibitory effects, which may be due to its relatively high abundance in the extracts and strong inhibitory potential compared with other alkaloids. Collectively, these observations suggest that more than one alkaloid is responsible for the total P450 inhibitory effects. The total inhibitory effect in vivo likely depends on alkaloid abundance in the product used, concentration at the enzyme site, and the inhibition kinetics of individual phytoconstituents.

Mitragynine has also been shown to inhibit UGT activity. UGT2B7 was inhibited in HLMs (IC_50_ ∼8 *μ*M), whereas UGT1A1 was unaffected (IC_50_ >100 *μ*M) (Abdullah and Ismail, 2018) (Table 4). However, the effects of kratom and its alkaloids on other major intestinal and hepatic UGTs, including UGT1A4, UGT1A6, UGT1A9, UGT1A10, UGT2B4, UGTB15, and UGT2B17, remain to be evaluated.

Mitragynine and 7-hydroxymitragynine were reported to inhibit the efflux transporter P-glycoprotein (P-gp). Both alkaloids inhibited P-gp–mediated efflux in MDR-MDCK cells and using the calcein-AM uptake assay, with an IC_50_ of ∼18 and 32 *μ*M, respectively (Manda et al., 2014a). Additionally, mitragynine (10 *μ*M) inhibited digoxin transport in Caco-2 cell monolayers to the same extent as the clinically relevant P-gp inhibitor quinidine (Rusli et al., 2019). High concentrations of mitragynine and other alkaloids attained in the intestinal lumen (∼400 *μ*M based on a 2-g kratom dose containing ∼40 mg mitragynine in 250 ml) could inhibit intestinal P-gp, attenuating the efflux of P-gp substrates to potentially increase oral bioavailability. Applying a basic static model, mitragynine was predicted to precipitate an interaction with P-gp (Table 5). Inhibition of another intestinal efflux transporter, breast cancer resistance protein (BCRP), has also been tested, but the IC_50_ (∼360 *μ*M) may be inconsequential (Wagmann et al., 2018). Effects of kratom on other drug transporters recognized to be of clinical importance by the FDA, including organic anion transporting polypeptides (OATPs), organic anion transporters (OATs), organic cation transporters (OCTs), multidrug resistance proteins (MRPs), and bile salt export pump (BSEP), are lacking (https://www.fda.gov/media/134582/download).

Kratom extracts and alkaloids, including mitragynine, have been reported to activate the pregnane X receptor in HepG2 cells (EC_50_ ∼5 *μ*g/ml and 1–5 *μ*M, respectively) (Manda et al., 2017; Hartley et al., 2022), which may induce the expression of certain drug metabolizing enzymes and transporters, including CYP3A and P-gp (Tolson and Wang, 2010). Although mitragynine did not increase CYP3A4 mRNA expression nor enzyme activity, the oxindole alkaloids isorotundifoline, isospeciofoline, corynoxine B, and corynoxine increased both markers by ∼2- to 3-fold relative to vehicle. However, given the typically lower abundance of these alkaloids compared with other well studied alkaloids in kratom products, the induction potential of kratom remains uncertain. Additional in vitro (e.g., human hepatocytes) and clinical studies, combined with physiologically based pharmacokinetic modeling and simulation, are warranted to determine the net effect of kratom-mediated time-dependent inhibition and induction of drug metabolism and transport upon chronic use.

**Fig. 3.**
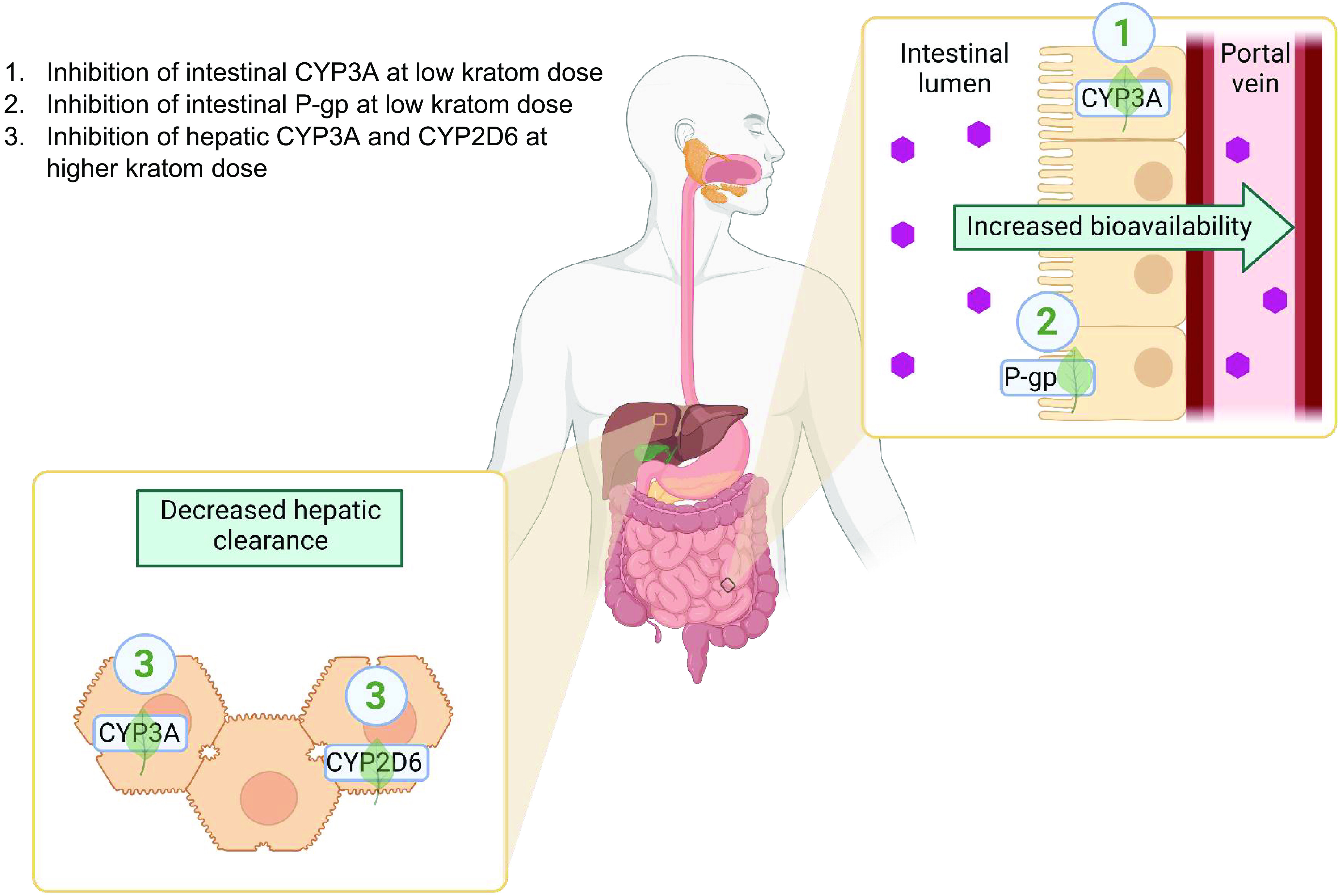
Identified targets and relevant mechanisms of potential pharmacokinetic kratom-drug interactions after oral administration of kratom (created with https://www.biorender.com/).

CYP3A and CYP2D6 metabolize >50% of marketed drugs (that are cleaved by oxidation) belonging to a broad range of therapeutic areas. Inhibition of these P450 enzymes could have major implications for pharmacotherapy. Specifically, both metabolize and eliminate several centrally acting drugs, including opioids and benzodiazepines (Mercadante, 2015). Thus, kratom users co-consuming such drugs may be at high risk of experiencing a pharmacokinetic/pharmacodynamic drug interaction (Fig. 3). Regarding conjugative enzymes, UGT2B7 is widely recognized as the major isoform responsible for metabolizing opioids, including morphine, codeine, some codeine metabolites, and other related compounds (Mercadante, 2015). These UGT2B7 drug substrates could also be involved in adverse drug interactions with kratom.

### Suspected Cases of Kratom-Drug Interactions in Humans

A recent report that compiled a total of 156 deaths associated with kratom use in the United States and Europe highlighted that 87% of the cases involved polysubstance use (Corkery et al., 2019). These cases were predominant in white (100%) males (80%) with a mean age of ∼32 years and a history of drug abuse (95%). Three cases that exemplify a suspected pharmacokinetic kratom-drug interaction are described.

*Case 1*. In Sweden, nine deaths within <1 year were linked to krypton (Kronstrand et al., 2011), which consists of kratom and *O*-desmethyltramadol, a metabolite of tramadol that is a more potent opioid than the parent drug. Postmortem blood samples from all nine decedents contained mitragynine and *O*-desmethyltramadol along with at least two or more other centrally acting drugs, including anxiolytics, antipsychotics, antidepressants, psychedelics, and sedatives. Pulmonary edema and bladder distension were the most commonly observed features postmortem, both of which are associated with opioid-like toxicity. These toxicities may be due to kratom/mitragynine, but the blood concentrations of mitragynine varied considerably (0.02–0.18 *μ*g/g). Because the observed toxicities were not directly correlated with mitragynine concentration, these effects may have been due to a toxic build-up of *O*-desmethyltramadol and/or other unknown opioids as part of the multidrug exposure. Kratom may have increased the systemic concentration of *O*-desmethyltramadol via inhibition of CYP3A4 or UGT2B7, contributing to overdose and death.

*Case 2*. A probable pharmacokinetic interaction between ingested kratom and the antipsychotic quetiapine was described for a 27-year-old male with a history of bipolar disorder, Asperger’s syndrome, and substance abuse (Hughes, 2019). He was found deceased with a toxic blood concentration of quetiapine (12 *μ*g/ml), which was ∼100-fold higher than therapeutic concentrations, along with valproic acid (8.8 *μ*g/ml) and qualitative detection of mitragynine. Based on pill count, the decedent was believed not to have ingested an excessive amount of quetiapine to attain the observed supratherapeutic blood concentration. Quetiapine is a substrate for CYP3A4 and P-gp, leading to suspicions of a pharmacokinetic kratom-drug interaction. Mitragynine-mediated time-dependent inhibition of intestinal and hepatic CYP3A4 activity could have diminished the first-pass metabolism of quetiapine via CYP3A4 (fraction metabolized ∼0.85), at least in the liver (fraction escaping gut metabolism ∼0.99) (Gjestad et al., 2017). Alternatively, mitragynine-mediated inhibition of intestinal P-gp could have increased the oral bioavailability of quetiapine, resulting in toxic systemic concentrations (Boulton et al., 2002).

*Case 3*. A 36-year-old male presented to the emergency department with serotonin syndrome and electrocardiogram abnormalities, which were suspected to result from adverse effects of the antidepressant venlafaxine and/or quetiapine, which were taken concurrently with an ultrahigh amount of kratom (∼90 g per day) (Brogdon et al., 2022). Kratom was speculated to inhibit the metabolism of either or both drugs, which is mediated by CYP2D6 and CYP3A, leading to drug build-up in the systemic circulation. Upon discontinuation of venlafaxine and quetiapine (but not kratom), the adverse effects gradually resolved. Reversal of these effects further supports that a kratom-drug interaction occurred.

In addition to pharmacokinetic mechanisms, some of the observed polyintoxications may have been exclusively pharmacodynamic in nature. For example, the presence of other drugs may lower the seizure threshold to increase the likelihood of kratom-mediated seizures. This mechanism may underlie the presumed interaction between kratom and the stimulant modafinil (Boyer et al., 2008), which is not a substrate for CYP2D6 or CYP3A nor does the drug cause seizures. However, modafinil, quetiapine, tricyclic antidepressants such as amitriptyline, and over-the-counter antihistaminic agents such as diphenhydramine and propylhexedrine are known to lower the seizure threshold (Holler et al., 2011; Umaharan et al., 2021). Accordingly, these drugs may increase the risk of seizures caused by kratom depending on the dose of kratom and the susceptibility of the user.

Overall, the information garnered from kratom-associated polyintoxications and the in vitro inhibitory effects of kratom (and its alkaloids) on P450 and P-gp activity suggests a high likelihood of observing additional pharmacokinetic kratom-drug interactions that affect drug pharmacodynamics. Mitragynine concentrations measured in postmortem blood or plasma (up to 12 *μ*M) approached or exceeded the inhibition constants (IC_50_, K_i_, K_I_) toward CYP2D6 and CYP3A (Papsun et al., 2019). The interaction risk may be higher for drugs that are extensively metabolized by intestinal CYP3A and/or are substrates of P-gp assuming a higher concentration of mitragynine in the intestine compared with the liver (Fig. 3). In addition, the overall risk of a pharmacokinetic kratom-drug interaction will depend on multiple factors, including kratom consumption pattern, coingested drugs, and the user’s genetic make-up.

## Perspectives and Path Forward

**Fig. 4.**
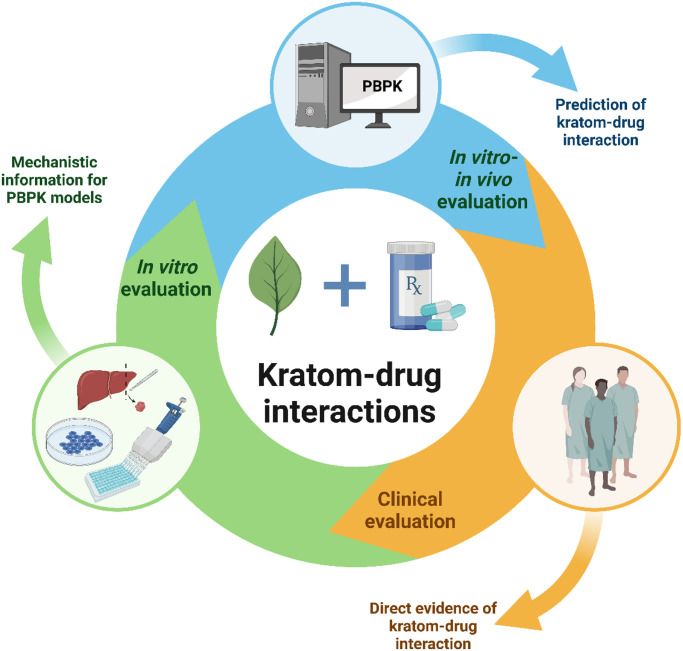
Proposed iterative approach for rigorous assessment of potential pharmacokinetic kratom-drug interactions (created with https://www.biorender.com/).

Increasing kratom use likely will continue due to the common (mis)perception that it is safe. The ready availability of kratom products and low cost make kratom the preferred treatment of pain and opioid withdrawal symptoms among kratom users (Coe et al., 2019). People often use kratom in an attempt to wean themselves from other dangerous drugs on the market, but they may relapse (White, 2018; Japarin et al., 2023). Hence, the likelihood of concomitant use of kratom with other psychoactive substances like opioids, benzodiazepines, or antidepressants is high. Taken together, thorough clinical evaluation of potential pharmacokinetic, as well as pharmacodynamic, kratom-drug interactions is warranted. The in vitro evidence and clinical case reports to date point to CYP3A, CYP2D6, and P-gp as targets of potential clinical pharmacokinetic kratom-drug interactions. An iterative approach involving physiologically based pharmacokinetic (PBPK) modeling and simulation and clinical pharmacokinetic studies can be used to comprehensively evaluate these interactions (Fig. 4).

### Clinical Assessment

Like pharmacokinetic drug-drug interactions recommended by regulatory agencies, kratom-drug interactions should be studied in a controlled clinical environment to obtain direct, actionable evidence. As mentioned earlier, clinical kratom-drug interaction studies are warranted for targets identified in this review, particularly CYP2D6, CYP3A, and P-gp. Studies should be conducted using well characterized kratom products and relevant probe drugs as described elsewhere for evaluating potential pharmacokinetic natural product-drug interactions (Kellogg et al., 2019; Cox et al., 2022). Evaluation of a well characterized kratom product representative of the majority of marketed products, administered in a form mimicking typical usage, will ensure generalizability of the results. An advantage of using probe drugs is that the results can be extrapolated to other drugs that either fully or partially rely on the corresponding metabolic or transporter pathways. The probe drugs can be administered at subtherapeutic doses, as the objective of the study is to observe a pharmacokinetic interaction, rather than a change in pharmacodynamics, in heathy participants. Chronic administration of kratom should also be considered because time-dependent inhibition of CYP3A was identified, which may be underestimated with a single dose.

Subsequent to studies involving probe drugs, pharmacokinetic interaction studies using clinically relevant object drugs causing a measurable change in pharmacodynamics can be undertaken. Pharmacodynamic changes can be measured using clinical biomarkers relevant to the object drugs. Clinical evaluation can later be expanded to other target drug metabolizing enzymes and transporters using larger probe cocktails (five to six object drugs), which may reveal other drug interactions that were either not tested or missed in vitro (Nguyen et al., 2021). Alternatively, emerging endogenous biomarkers such as coproporphyrins for OATPs, *N*-methyl nicotinamide for OCTs, and homovanillic acid/pyridoxic acid for OATs (Li et al., 2021) can be monitored for alteration in transporter activity with kratom administration.

### PBPK Modeling and Simulation

As for natural product-drug interactions in general, the complexities involved in assessing kratom-drug interactions, including the compositional variability of marketed kratom products and diverse consumption patterns, can be overcome using in vitro to in vivo extrapolation (IVIVE) approaches (Cox et al., 2021). Dynamic mathematical models, including PBPK models, which consider both human physiology and drug-related parameters, have been extensively used to support the drug discovery and development process. PBPK models can be applied to kratom-drug interactions to simulate various scenarios that are otherwise difficult or unethical to test in humans. Such model predictions are increasingly accepted by regulatory agencies in lieu of results generated from formal clinical pharmacokinetic interaction studies. The available mechanistic in vitro information about the P450 and P-gp inhibitory effects of kratom and the clinical pharmacokinetics of key kratom alkaloids using a well characterized product can be used to develop robust PBPK models. The next steps in this iterative approach (Fig. 4) would be to clinically assess potential pharmacokinetic kratom-drug interactions, the data from which can be used to refine the PBPK models, potentially prompting follow-up clinical studies. Unforeseen observations from clinical studies can also prompt follow-up clinical studies. The end goal would be to continue refining the PBPK model with these additional clinical, as well as mechanistic in vitro, data to accurately simulate various real-world scenarios (e.g., different object drugs, kratom doses, kratom consumption patterns, special populations).

## Summary

The research community is beginning to fill critical knowledge gaps regarding the epidemiology, chemistry, pharmacology (pharmacokinetics and pharmacodynamics), and toxicology of kratom. The increasing number of kratom-related adverse events involving polyintoxication warrants a thorough investigation of potential kratom-drug interactions, which can be accomplished using an iterative approach involving additional mechanistic in vitro, PBPK modeling and simulation, and clinical assessments. The widespread availability and use of kratom products underscore the urgency of this research. Multidisciplinary collaborations among the natural products industry, academia, and government agencies are essential to reduce the time lag in providing this essential information. National Institutes of Health National Center for Complementary and Integrative Health, National Institute on Drug Abuse, and other agencies are funding several research projects to create awareness among kratom users, healthcare providers, and federal regulators. Results from these projects will help various authorities develop scientifically informed policies to promote the safe use of kratom and mitigate further public health hazards.

## Abbreviations

AUC: area under the concentration versus time curve
DEA: US Drug Enforcement Administration
FDA: US Food and Drug Administration
F_oral_: absolute oral bioavailability
hCES: human carboxylesterase
HIM: human intestinal microsome
HLM: human liver microsome
*K_I_*: time-dependent inhibition constant
*K_i_*: reversible inhibition constant
*k_inact_*: maximum rate of inactivation
LCMS: liquid chromatography–mass spectrometry
P450: cytochrome P450
PBPK: physiologically based pharmacokinetic
P-gp: P-glycoprotein
UGT: UDP-glucuronosyltransferase
